# Experimental phasing: best practice and pitfalls

**DOI:** 10.1107/S0907444910006335

**Published:** 2010-03-24

**Authors:** Airlie J. McCoy, Randy J. Read

**Affiliations:** a Cambridge Institute for Medical Research, University of Cambridge, Hills Road, Cambridge CB2 OXY, England

**Keywords:** enantiomers, handedness, absolute configuration, chirality, twinning, experimental phasing

## Abstract

Developments in protein crystal structure determination by experimental phasing are reviewed, emphasizing the theoretical continuum between experimental phasing, density modification, model building and refinement. Traditional notions of the composition of the substructure and the best coefficients for map generation are discussed. Pitfalls such as determining the enantiomorph, identifying centrosymmetry (or pseudo-symmetry) in the substructure and crystal twinning are discussed in detail. An appendix introduces com­bined real–imaginary log-likelihood gradient map coefficients for SAD phasing and their use for substructure completion as implemented in the software *Phaser*. Supplementary material includes animated probabilistic Harker diagrams showing how maximum-likelihood-based phasing methods can be used to refine parameters in the case of SIR and MIR; it is hoped that these will be useful for those teaching best practice in experimental phasing methods.

## Abstract 2

The pitfalls of experimental phasing are described.

## 1. Introduction

Experimental phasing of protein structures is usually (although not always) a more difficult and time-consuming process than phasing a protein structure by molecular replacement. Experimental phasing is required when there is no sufficiently good template for molecular replacement, which is the case when studying proteins with no (or low) sequence identity to proteins for which the structure is known; that is, proteins with new (or very different) folds. Since these structures tend to provide a wealth of novel biological information, experimental phasing remains a key tool in the crystallo­grapher’s toolkit.

The theory and practice of experimental phasing is covered in all protein crystallography text books (including Blundell & Johnson, 1976 ▶; Drenth, 1994 ▶; Blow, 2002 ▶), in online resources (including our website at http://www-structmed.cimr.cam.ac.uk/Course) and in journal articles (including, in this issue, Taylor, 2010 ▶). This paper assumes a basic understanding of experimental phasing and aims to point out the state-of-the-art methodologies and shed light on some of the more tricky aspects of the process.

## 2. Substructures

The phasing process starts with finding a few atoms (or even a single atom) in the asymmetric unit of one of the crystals from which data have been collected. The initial set of atoms is found using Patterson, direct methods or dual-space methods [implemented in software such as *HySS* (Grosse-Kunstleve & Adams, 2003*a* ▶), *Shake-and-Bake* (*SnB*; Miller *et al.*, 1994 ▶) and *SHELXD* (Sheldrick, 2008 ▶)]. The set of atoms is called a ‘substructure’, simply because it is a subset of the atoms in the full structure. The substructure is usually thought of as all the atoms in the molecule that are not carbon, nitrogen, oxygen or sulfur (or phosphate for nucleic acids), such as anomalously scattering or heavy atoms deliberately added to the crystals or fortuitous intrinsic metal ions. However, this concept of the substructure does not reflect current phasing practice. Any set of atoms, up to and including the full structure, can be con­sidered a ‘substructure’. In particular, for a single-wavelength anomalous dispersion (SAD) experiment the substructure need not only include atoms that have significant anomalous scattering and for a single-wavelength anomalous dispersion (SIR) experiment the substructure need not only include atoms that are heavy; in both cases C, N and O atoms can also be part of a substructure. Thus, a partial molecular-replacement solution is also a valid initial sub­structure. Inclusion of minor sites improves the phases because the more complete the substructure, the better the phases; in the limit, the best phases are calculated from the complete structure. Including ‘minor’ sites in the phasing is important because what they lack in individual scattering they can make up for in total scattering as a group. Experimental phasing can be considered as a process of bootstrapping from a tiny substructure to an almost complete substructure (raising the question: is the model ever complete?).

Substructure atoms found independently in different derivatives need not have the same hand or be on the same origin for the space group. If multiple-wavelength isomorphous replacement (MIR) or MIR with anomalous scattering (MIRAS) phasing is undertaken with the sites in different derivatives having different hands (see section §[Sec sec6]6 below) or on different origins then the phasing will fail. To make sure that the hands and origins of all the sites in all the derivatives are consistent, one derivative is chosen as the reference (usually the first derivative for which a substructure has been determined, unless this derivative has centrosymmetry; see §[Sec sec7]7 below) and difference Fourier maps (Stryer *et al.*, 1964 ▶; chapter 14 of Blundell & Johnson, 1976 ▶) or log-likelihood gradient maps (Von­rhein *et al.*, 2007 ▶; Appendix *A* [App appa]) are used to find a substructure for the other derivatives. Indeed, this is usually the fastest way of finding a substructure for the other derivatives, especially if the anomalous or isomorphous signal in the other derivatives is not as good as for the reference derivative.

## 3. Phasing

**Figure 1:**
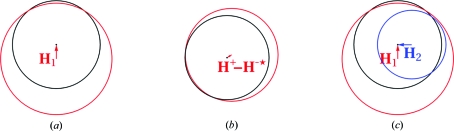
Harker diagrams. (*a*) SIR Harker diagram where **H**_1_ is the calculated substructure structure factor for the single derivative. The black and red circles have radii given by the observed structure-factor amplitudes for the native and the derivative, respectively. (*b*) SAD Harker diagram where **H** ^+^ and **H** ^−^ are the calculated substructure structure factors and **H** ^+^ − **H** ^−*^ is the expected vector difference between the true structure factors** F** ^+^ and **F** ^−*^. (*c*) MIR Harker diagram where **H**_1_ and **H**_2_ are the calculated substructure structure factors for the first and second derivatives, respectively. The black, red and blue circles have radii given by the observed structure-factor amplitude for the native, the first derivative and the second derivative, respectively. In the absence of measurement errors and errors in the substructure, the red and blue circles would intersect at one point on the black circle.

There is a phase ambiguity in SIR and SAD which is clearly shown on a Harker diagram (Figs. 1 ▶ *a* and 1 ▶ *b* and Supplementary Figs. S1*a* and S1*b* 1). The correct set of phases gives the true electron-density map and the incorrect set gives noise (Wang *et al.*, 2007 ▶). It is not possible to generate and inspect maps for all possible combinations of phases to resolve the phase ambiguity; the number of combinations is a ‘lifetime-of-the-universe’ size problem. Instead, maps are calculated with the average of the two possible phases for each structure factor (Blow & Rossmann, 1961 ▶). This is a good approximation to the correct phase when the two phase possibilities are close together and becomes poorer as the two phase possibilities move to being 180° apart. The map calculated with the average of the two phases is the true electron density plus noise, *i.e.* the superposition of the map calculated with the true phases and the map calculated with the wrong phases.

The noise can be removed from the map (or at least reduced) with density-modification methods. Density modification has the effect of selecting the correct phase from the two phase possibilities. Thus, in the case of SAD and SIR the improvements in the map can be very dramatic. Traditional density-modification methods include solvent flattening (Wang, 1985 ▶) or flipping (Abrahams & Leslie, 1996 ▶), histogram matching (Zhang & Main, 1990 ▶) and noncrystallographic symmetry averaging (Rossmann & Blow, 1963 ▶, 1964 ▶). More recently, and, in particular, since the development of automated model-building algorithms, model building has become part of the density-modification process; model building can be thought of as the most drastic type of density modification.

A second experimental source of phase information also breaks the phase ambiguity inherent in SAD and SIR (Blundell & Johnson, 1976 ▶, p. 160, p. 180 and references therein). In a purely isomorphous replacement phasing experiment (MIR) the minimal requirement for a unique phase determination is two derivatives (and a native). In a purely anomalous scattering experiment (multiwavelength anomalous dispersion; MAD) the minimal requirement is data that have been collected at two different wavelengths. Iso­morphous replacement and anomalous scattering can also be combined in SIR with anomalous scattering (SIRAS) or MIRAS experiments to give a unique phase.

Some real Harker diagrams from the phasing of haemoglobin with six derivatives [Cullis *et al.*, 1961 ▶; reproduced on p. 367 of Blundell & Johnson (1976) and in Fig. 7.22 of Blow (2002)] ▶ show that despite extremely well determined data the phase circles in these examples do not cross exactly. Unfortunately, these sorts of Harker diagrams are not exceptional and the true phase is often only poorly indicated even with the addition of more derivative data.

**Figure 2:**
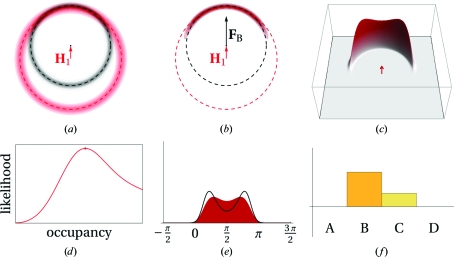
SIR probabilistic Harker diagram (notation as in Fig. 1 ▶). (*a*) Contour plot showing components of the PDF. The component arising from the native is shown in black contours and the component arising from the derivative is shown in red contours centred on **H**_1_ (the point at the base of the red arrow). The dashed black and red circles indicate the measured values of the observed structure-factor amplitudes for the native and the derivative, respectively. (*b*) The PDF [the product of the two components in (*a*)] is shown in dark red contours. The ‘best **F**’ **F**_B_ is shown as a black arrow. (*c*) Three-dimensional plot of the value of the PDF. The likelihood is the volume under the PDF surface. (*d*) Plot of the likelihood as a function of the occupancy of the substructure (increasing amplitude of **H**_1_). The maximum likelihood is marked with a dot. All other panels in this figure show the values of the parameters at the point of maximum likelihood. (*e*) The PDF for the phases of the true structure factor **F** is shown in red and the PDF reconstructed from the four Hendrickson–Lattman (Hendrickson & Lattman, 1970 ▶) coefficients (HL) is shown as a black curve. (*f*) Bar chart showing the relative values of the four HL coefficients *A*, *B*, *C* and *D*.

The problem of non-overlapping Harker circles in MIR (Fig. 1 ▶ *c* and Supplementary Fig. S1*c* 1) was initially approached by using a parameter for the geometrical lack of closure of the phase triangle (Blow & Crick, 1959 ▶; see Blundell & Johnson, 1976 ▶, p. 366). A better approach is to use the probabilistic Harker construction and maximum likelihood to find the phase (for a review, see McCoy, 2004 ▶). Instead of a single circle for each structure factor there is a circular probability distribution obtained by ‘smearing out’ the Harker circles with a Gaussian distribution. The product (multiplication) of the individual probability density functions for each data set gives a combined probability density function (PDF) for the true structure factor (Figs. 2 ▶, 3 ▶ and 4 ▶).

In the probabilistic approach it is possible to optimize (refine) the substructure parameters, which are not well determined by the initial substructure-location programs. Although the positions of the substructure atoms are relatively well determined, the occupancies are only poorly estimated from the relative Patterson peak heights (some algorithms do not even attempt to make an estimate but simply output an equal occupancy of 1 for each of the sites they find). Individual atomic *B* factors cannot be estimated, so all *B* factors are either set to an arbitrary constant value (*e.g.* 20 Å^2^) or to the Wilson *B* factor of the data. The scattering factors *f*′ and *f*′′ can be estimated from the values given in the Sasaki tables (Sasaki, 1989 ▶), which tabulate *f*′ and *f*′′ values for the elements against wavelength. These values are only good for initial estimates because they are calculated assuming ‘free’ atoms, while the anomalous scatterers in the crystal are in chemical bonds which alter the resonances. Alternatively, *f*′ and *f*′′ can be determined experimentally by carrying out a fluorescence scan (Evans & Pettifer, 2001 ▶). There is also another important class of parameters to refine: the estimates of the errors of the parameters (variances) of the PDF. To refine the parameters (position, occupancy, *B* factor, scattering factors and variances), the area under the PDF curve (the integral of the PDF) is optimized (Figs. 2 ▶, 3 ▶ and 4 ▶, and Supplementary Figs. S2, S3 and S4^1^).

Likelihood methods are good for refining the substructure because they account for errors in the model and the data. However, this is only true when the errors are not systematic errors, *i.e.* when the error model used in the derivation of the likelihood function correctly models the sources of error in the experiment. Errors that derive from, for example, non-isomorphism and radiation damage are not part of the error model and will degrade the quality of the phases. Where non-isomorphism and/or radiation damage is present it is important to optimize the set of data sets used in phasing and/or to exclude data at high resolution (where the errors will be greatest). An example of this was presented at the 2003 CCP4 Study Weekend on the topic of Experimental Phasing (Evans, 2003 ▶).

## 4. Calculating electron density

Electron density is calculated using the electron-density equation, which is the Fourier transform of the structure factors,
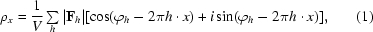
where ρ is the electron density, *x* represents the spatial co­ordinates (*x*, *y*, *z*), *V* is the volume of the unit cell, *h* represents the reciprocal-space indices (*h*, *k*, *l*), |**F**_*h*_| is the amplitude of the structure factor and ϕ_*h*_ is the phase of the structure factor **F**_*h*_. Note that if Friedel’s law applies and |**F**_*h*_| = |**F**_−*h*_| and ϕ_*h*_ = −*ϕ*_*h*_ (*i.e.* the diffraction pattern has a centre of inversion at the origin) then the sine terms for *h* and −*h* cancel and the imaginary component is zero everywhere; the electron density is real. If Friedel’s law does not apply then the imaginary term is not zero. The imaginary component can be represented as a second real electron-density map. The peaks in this second map are the positions of the anomalously scattering atoms that cause Friedel’s law to break down.

What structure factor should be used in the electron-density equation in the probabilistic approach? We have to pick one phase and amplitude for substitution into the electron-density equation. The best structure factor will usually be the one that gives the lowest root-mean-square deviation between the calculated electron density and the true electron density. (If there are sources of model bias, for instance the real scattering contribution from the anomalous scatterers in SAD phasing, then it may be preferable to include a bias correction). Parseval’s theorem (of Fourier transforms) relates the root-mean-square error in real space to the root-mean-square error in reciprocal space and *vice versa*. Using this theorem, it can be shown that the best structure factor (**F**_best_) is the ‘centroid’ structure factor (the probability-weighted average of all the structure factors); it is not the ‘most probable’ structure factor (Fig. 5 ▶). The amplitude of **F**_best_ is always less than *F*_obs_ (always inside the circle of the Harker diagram; Figs. 2 ▶, 3 ▶ and 4 ▶, and Supplementary Figs. S2, S3 and S4). The reduction in *F*_obs_ to give |**F**_best_| is expressed as the figure of merit (*m*, where 0 ≤ *m* ≤ 1; *m* = 1 implies perfect phases and *m* = 0 implies no phase information). The probabilistic approach puts the approximation of taking the average of the two phases for map calculation in the case of SAD and SIR onto a firm theoretical footing. It has the added advantage of showing how to up-weight the structure factors (high figure of merit) when the two possible phases are close together and down-weight the structure factors (low figure of merit) when the phases are further apart.

The probabilistic approach thus shows that maps with co­efficients *mF*_obs_ have the lowest noise. When the model is ‘nearly complete’, that is, the calculated structure factors are good approximations to the true structure factors and the phase error is low, then the map with coefficients* mF*_obs_ shows electron-density features that are present in the true structure but missing from the model at half-weight. To boost the peaks of the electron density at the places where the model is incomplete, crystallographers and model-building algorithms usually look at maps with coefficients 2*mF*_obs_ − *DF*_calc_ (where *D* is a value between 0 and 1; Read, 1986 ▶) during refinement. These coefficients double the *mF*_obs_ map (thus bringing the unmodelled features up to full weight) and subtract one copy of the model, but at the expense of doubling the noise. In cases where the real scattering of the substructure is a significant fraction of the true structure factor, 2*mF*_obs_ − *DF*_calc_ maps may also be useful in experimental phasing before model building starts.

## 5. Handedness

Compounds such as proteins that are not superimposable on their mirror images are chiral compounds. The chiral arrangement of atoms is also known as the ‘absolute configuration’, the ‘enantiomer’ and, more colloquially, the ‘hand’ of the compound. Naturally occurring proteins consist of l-amino acids (*i.e.* left-handed amino acids) and right-handed α-helices, but a small number of proteins consisting of d-­amino acids and left-handed α-helices have successfully been synthesized and their structures solved (Pentelute *et al.*, 2008 ▶). The handedness of amino acids can be remembered using the ‘CORN law’ (Blundell & Johnson, 1976 ▶, pp. 18–19). The amino acid can be thought of as a tetrahedron placed on a horizontal surface with the C^α^ atom at the body centre and its H atom pointing upwards. Then, for l-amino acids the α-carbonyl CO group, the side chain *R* group and the α-amino N group are located clockwise around the base of the tetrahedron; for d-amino acids the CO-*R*-N groups are located anticlockwise.

The handedness of the protein can be determined from the diffraction pattern when there is significant anomalous scattering and thus Friedel’s law is broken (Bijvoet, 1949 ▶, 1954 ▶). If there is only normal scattering and the intensity of reflection (*h*, *k*, *l*) is equal to the intensity of reflection (−*h*, −*k*, −*l*) then the diffraction cannot show the hand: a structure and its mirror image fit the data identically.

Tracking the hand of the protein through the diffraction experiment is nontrivial. The diffraction from either hand can be worked out from first principles using the Laue equations and the 90° phase lag of the anomalous scattering with respect to the incoming wave (Blundell & Johnson, 1976 ▶, p. 167; James, 1957 ▶, pp. 35–36). This anomalous scattering is thus 90° phase-advanced with respect to the normally scattered wave (which is 180° out of phase with the incoming wave); the anomalous structure factor is thus drawn 90° anticlockwise (*i.e* advanced) from the normally scattering component on a Harker diagram (Fig. 6 ▶). The coordinate system for the atoms (*x*, *y*, *z*) and the coordinate system for the reciprocal lattice (*h*, *k*, *l*) are both conventionally right-handed. There is a tricky step at the stage of the Fourier transform used to generate the electron density. Crystallographers use the forward Fourier transform to calculate structure factors and the inverse Fourier transform to calculate electron density. The inverse Fourier transform uses (−*x*, −*y*, −*z*), which is a change-of-hand operation. If all these operations are kept track of correctly, then the Friedel differences will show l-amino acids for naturally occurring proteins.

Unfortunately, the Friedel diffraction information that can determine the hand is lost when initially determining the substructure by Patterson methods or so-called ‘direct methods’. These methods only use the magnitude of the anomalous difference |*F* ^+^ − *F* ^–^|. As we shall see, it is the direction of the anomalous difference that is important in determining the hand, *i.e.* whether *F* ^+^ > *F* ^−^ or *vice versa*. In addition, initial substructures found by substructure-location programs contain only one type of atom and so the calculated structure factors do not have a Friedel difference (see discussion below). Therefore, the hand of the initial substructure is arbitrary; both sets of sites satisfy the anomalous differences (whether through Patterson or ‘direct methods’) equally well. Part of the process of the diffraction experiment is to find which hand of the substructure is correct, *i.e.* is con­sistent with l-amino acids. (Note that if a partial molecular-replacement solution is used as the initial substructure then the hand is correct by virtue of the molecular-replacement model having the correct hand.)

For nonchiral space groups (except for *I*4_1_, *I*4_1_22 and *I*4_1_32), the substructure is converted to its other hand by the inversion operation through the origin (*x*, *y*, *z*)→(−*x*, −*y*, −*z*). For chiral space groups, in addition to inverting the coordinates of the substructure through the origin, the space group must also be changed to its chiral partner (Table 1 ▶). For the three non­chiral space groups *I*4_1_, *I*4_1_22 and *I*4_1_32 the other hand of sites is not obtained using simple inversion through the origin. These space groups are exceptions because they ‘should’ have chiral pairs (*I*4_3_, *I*4_3_22 and *I*4_3_32, respectively); however, the crystallographic symmetry of these space groups (in particular, the body centring) generates a 4_3_ screw from the 4_1_ screw operation (and *vice versa*). Thus, the chiral partners for these three space groups that ‘should’ exist are not distinct space groups. By convention (*International Tables for Crystallo­graphy*, 2002 ▶), the space groups are defined with a 4_1_ screw axis and so only space groups *I*4_1_, *I*4_1_22 and *I*4_1_32 ‘exist’. Because of this convention, inverting the substructure requires the inversion operation through the origin (*x*, *y*, *z*)→(−*x*, −*y*, −*z*) followed by shifting the sites in the unit cell to position them around the alternate screw symmetry axis. Alternatively, in these three space groups the change-of-hand operation can be considered to be an inversion through a point that is not the origin.

The inverse hand of the substructure gives different Harker diagrams for SAD and SIR phasing (see Figs. 2 and 4 in Wang *et al.*, 2007 ▶) and electron density with different features. For SIR, the other hand gives a Harker diagram reflected through the real axis of the Argand diagram. The other phase gives the mirror-image density. Density-modification methods that do not involve model building give equally good statistics in both hands; only by model building can the correct hand be identified. For SAD, the other hand gives a Harker diagram reflected through the imaginary axis of the Argand diagram. If the contribution from the real scattering from the substructure is neglected, the other phase gives the mirror-image density in negative (peaks become holes). Density modification is better in the correct hand and the hand can be determined before model building from the density-modification statistics.

Under certain circumstances (that is, if the substructure has special properties) the hand can be found with anomalous differences even without density modification. To understand this, consider the case at the end of refinement when there is a good model for the structure (the ‘substructure’ is almost the ‘true’ structure). If there are anomalous differences, then there are anomalously scattering atoms in the model and the calculated structure factors have a Friedel difference between *F* ^+^_calc_ and *F* ^−^_calc_, *i.e. F* ^+^_calc_ ≠ *F* ^−^_calc_ (Fig. 6 ▶). For example, in a case with a perfect model and perfect data, if hand *A* has *F* ^+^_calc_ = 42 and *F* ^−^_calc_ = 39 so that *F* ^+^_calc_ > *F* ^−^_calc_, then hand *B* will have *F* ^+^_calc_ = 39 and *F* ^−^_calc_ = 42 so that *F* ^+^_calc_ < *F* ^−^_calc_. Only in one hand will *F* ^+^_calc_ and *F* ^−^_calc_ match the observed values, *e.g.* if *F* ^+^_obs_ = 42 and *F* ^−^_obs_ = 39 then hand *A* would be correct. In the ideal case, the matching of the Friedel difference would be true for all reflections. With imperfect data and an imperfect model, one hand will be more successful in predicting the direction of the observed anomalous difference (*F* ^+^_obs_ > *F* ^−^_obs_ or *vice versa*) over all the reflections and this statistical bias will indicate the correct hand. Therefore, it is possible to discover the hand from the anomalous differences alone (*i.e.* without inspecting the electron density) whenever the structure factors calculated from the substructure have Friedel differences. Unfortunately, this is not the case if the substructure consists of only one type of anomalous scatterer. For example, if the substructure consists of only the selenium sites of a selenomethionine protein then the substructure cannot predict the hand. (As an aside, a real crystal consisting of a single type of anomalous scatterer also has no Friedel difference; diffraction from crystals of mineral selenium does not have a Friedel difference.) For the calculated structure factors to have a Friedel difference, the substructure must have more than one scattering type, at least one of which must be a significant anomalous scatterer (Fig. 6 ▶). (More exactly, the ratio of the normal and the anomalous components of all the structure factors of the atoms in the substructure must not all be the same, so that the anomalous component of the calculated structure factor is not perpendicular to the normal scattering.)

Thus, with SIR and MIR, and any number of scatterers, the parameters of the model need only be refined with the sub­structure in one hand; the other hand can be phased using the refined parameters. The correct hand is found by inspecting the density (*i.e.* by model building, finding which hand of the peptide or nucleotide fits the electron density). For any experimental phasing method that includes an anomalous difference (*e.g.* SAD, SIRAS, MAD and MIRAS), if there is only one type of (anomalous) scatterer in the substructure then only one hand need be refined (however, if both hands are refined it is unlikely that the phasing statistics will be identical, simply because of different rounding errors in the computations). The other hand can be phased from the refined parameters from the first hand and density-modification statistics can be used to determine the correct hand. If there are two or more types of scatterer (one of which must have significant anomalous scattering) in the substructure then the substructure parameters must be refined in both hands. The correct hand can be determined from the phasing statistics, since one hand will fit the observed direction of the anomalous differences in the data better than the other hand.

Other methods have been used for determining the hand. Blundell & Johnson (1976 ▶) suggest two ways of obtaining the hand by SIRAS. The first method (p. 181) is to calculate the imaginary part of the anomalous difference Fourier for phases obtained using the isomorphous information only (*i.e.* SIR). If the hand is incorrect then ‘the Fourier gives rise to negative holes at loci which are related by inversion through the origin to the anomalous scatterer.’ This is equivalent to looking at the SIRAS-phased electron density and finding mirror-image density in negative electron density, but is easier to identify by eye (the only method available in 1976) as the imaginary map is less noisy than the real map. The second SIRAS method (p. 182) involves calculating the phases twice ‘by combining isomorphous and anomalous scattering data once for each heavy-atom configuration’ and then inspecting the density for ‘recognisable features’. If more than one isomorphous derivative is available then Blundell & Johnson (1976 ▶) suggest (p. 182 and 375; see also §9.4 of Drenth, 1994 ▶) that the hand is distinguished by using the two phase sets in isomorphous difference Fourier syntheses to find the location of the heavy atoms in the second derivative. The correct hand then ‘should give phases leading to the largest peak’ in the difference Fourier because the density at the heavy-atom locations ‘will be reinforced when the anomalous scattering information is included with the correct hand and diminished when the hand is wrong’. These two methods are equivalent to using density-modification statistics, as they involve inspecting electron density to find the better of the two maps.

## 6. Centrosymmetric sites

Occasionally (but more often than one would like) the distribution of anomalous or heavy atoms in the substructure is centrosymmetric. If the space group is *P*1, then a sub­structure of one or two identical atoms will always be centrosymmetric. Atoms on special positions are often centrosymmetric (for example, the two Zn atoms in 2Zn insulin on the threefold axis of space group *R*3; Blundell *et al.*, 1972 ▶). Other unfortunate distributions of atoms in combination with the space-group symmetry may also be centrosymmetric. When the sites are centrosymmetric, structure solution is more difficult.

Centrosymmetric substructures in SAD and SIR result in electron-density maps with very different properties to those calculated with noncentrosymmetric substructures. Recall that SAD and SIR give a phase ambiguity and that an electron-density map calculated with the average of the two possible phases is the superposition of the true electron density and ‘noise’. In SIR the ‘noise’ is the mirror image of the true electron density convoluted with the Fourier transform of exp(2*i*ϕ_sub_), where ϕ_sub_ are the phases of the substructure. This map looks random for a noncentrosymmetric substructure. In SAD the ‘noise’ is the negative inverse of the true electron density convoluted with the Fourier transform of exp(2*i*ϕ_sub_), which also looks random for a noncentrosymmetric sub­structure. However, if the substructure is centrosymmetric then all the substructure phases are either 0 or π and thus exp(2*i*ϕ_sub_) = 1 and the ‘noise’ map does not look random. The SIR map becomes a superposition of the true electron density with its mirror-image density and the SAD map becomes the superposition of the true electron density with its mirror-image density in negative. Note that these maps have the same form as the maps calculated using the two hands of the sub­structure (as expected, since the centrosymmetric substructure can be thought of as having ‘both hands at the same time’). Interpreting the maps thus becomes much more difficult as there are features above the noise level that are not attributable to the true electron density.

It is often not immediately obvious that a substructure is centrosymmetric. A simple geometrical approach to the problem (*i.e.* inspecting the coordinates) will find atoms that are related by inversion through the origin. For exact centrosymmetry, all atoms must have a centrosymmetric partner. Since it is the scattering from the atoms that is the issue, another condition of exact centrosymmetry is that the *B* factors and occupancies of the atoms at positions inverted through the origin must be identical. However, it is highly unlikely that all the atomic parameters will be exactly centrosymmetric and the more the centrosymmetry is broken the less difficult structure solution will be. The disadvantage of the simple geometric approach is that it is unable to quantify how difficult a pseudo-centrosymmetric arrangement will make structure solution or how difficult structure solution will be when only a subset of the sites is centrosymmetric. The *phase-o-phrenia* algorithm (Grosse-Kunstleve & Adams, 2003*b* ▶) goes to the heart of the problem and in effect looks at how closely the substructure phases are clustered around 0 and π. In order to avoid problems with the three space groups in which the centre of inversion is not at the origin (in which case the phases are π apart but not 0 and π) the algorithm actually looks at how closely the Fourier transform of exp(2*i*ϕ_sub_) resembles a delta function (since the Fourier transform of a constant value is a delta function). The *phase-o-phrenia* plot for one randomly placed atom in *P*1 generates a ‘δ-function’ plot clearly showing the centrosymmetry of this substructure. Conversely, four randomly placed atoms in *P*31 generate a ‘flat’ plot and therefore are not centrosymmetric. The *phase-o-phrenia* algorithm also shows that some maps will be more difficult to interpret than others even if the substructure is not centrosymmetric. For example, one randomly placed atom in *P*3 gives a *phase-o-phrenia* plot that is close to that of a δ-­function, because the substructure has 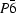 symmetry with a mirror plane passing through the atom.

If the substructure for the reference structure has centrosymmetry (or pseudosymmetry) then difference Fourier maps for other derivatives will also have this higher symmetry, since the centrosymmetry (or pseudosymmetry) is encoded in the phases. Difference Fourier maps calculated with these phases will show fallacious high peaks which can be mistaken for real atoms. To avoid this problem, only one peak should be selected from the difference Fourier in the first instance and the computation of the phases should be repeated with the additional site. In this way, new sites will be consistent with one choice of hand. However, in our experience it can be very difficult to break the centrosymmetry by only adding one site in a new derivative at a time and it can be better to find the sites in the new derivative independently and then use this derivative as the reference for locating the substructure in other derivatives.

## 7. Twinning

Twinning (of the merohedral or pseudo-merohedral type; Parsons, 2003 ▶) makes experimental phasing particularly difficult. The problems lie both in finding an initial substructure and interpreting the (twinned) electron density. Those crystals where structure solution has been successful were phased by either ignoring the twinning entirely (if the twin fraction α was very low) or using the technique of ‘detwinning’ the data (*i.e.* estimating the untwinned intensities from the observed structure-factor intensities). Twinned protein structures have been solved using a range of experimental phasing methods: SIR (Declercq & Evrard, 2001 ▶), MIR (Terwisscha van Scheltinga *et al.*, 2001 ▶), MIRAS (Ban *et al.*, 2000 ▶) and MAD (Rudolph *et al.*, 2003 ▶; Dauter, 2003 ▶). Structure solution by experimental phasing is possible even when there are more than two components of the twinning (Barends *et al.*, 2005 ▶). Unfortunately, the detwinning method is only applicable when the twin fraction is not too close to 0.5, because as the twin fraction increases errors in the estimation of the detwinned intensities rise dramatically [the variances are proportional to the term (1 − 2α)^−2^]. Because of the errors introduced by the detwinning, successful phasing requires that errors from other sources be reduced as much as possible; success generally requires better measured data with stronger anomalous and/or isomorphous signals than would be required for untwinned crystals. To minimize the errors from the detwinning, structure determination invariably involves screening many native and derivative crystals in order to find those with the lowest twin fractions.

A theoretical framework which does not rely on detwinning the intensities has been described for MIR phasing of (two-component) twinned data in the general case, including perfectly twinned data (Yeates & Rees, 1987 ▶). This method can be visualized as extending the two-dimensional Harker diagram into four dimensions, with the Harker circles becoming four-dimensional hyper-spheres. Four derivatives are necessary to uniquely determine the phase rather than two for conventional MIR.

In our experience with the *Phaser* software (McCoy *et al.*, 2007 ▶), it is common to solve structures of high or perfect twins by molecular replacement (although the template structure needs to represent the target structure more accurately than for nontwinned crystals) and so an alternative approach could be to solve (or find in the database) the structure of a related protein for use as a template for molecular-replacement trials. Once there is a molecular-replacement solution, even if it is not good enough to enable model building and refinement, we have found that log-likelihood gradient map completion (see Appendix *A* [App appa]) can succeed in finding the anomalous scatterers from twinned SAD data, which can then be used to improve the phases.

## 8. Conclusion

The development of automated pipelines (Adams *et al.*, 2002 ▶, 2004 ▶; Brunzelle *et al.*, 2003 ▶; Lamzin & Perrakis, 2000 ▶; Lamzin *et al.*, 2000 ▶; Panjikar *et al.*, 2005 ▶; Pape & Schneider, 2004 ▶; Snell *et al.*, 2004 ▶, Vonrhein *et al.*, 2007 ▶) means that, at least in straightforward cases, it is possible to build an atomic model of a protein structure using experimental phasing without the need for manual intervention. In these pipelines, problems such as hand determination are carried out silently without the need for users to even know that the problem exists. However, pathologies such as centrosymmetry and twinning will require manual intervention for the foreseeable future and in these cases it is vitally important to be aware of the potential pitfalls, since the outcome of even a simple misstep can be catastrophic (Chang *et al.*, 2006 ▶).

## Supplementary Material

Animated version of Fig. 1.. DOI: 10.1107/S0907444910006335/ba5142sup1.gif

Animated version of Fig. 2.. DOI: 10.1107/S0907444910006335/ba5142sup2.gif

**Figure 3:**
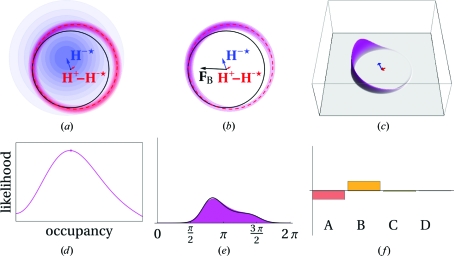
SAD probabilistic Harker diagram (adapted from McCoy, 2004 ▶ with notation as in Fig. 1 ▶). (*a*) Contour plot showing components of the PDF. The component *P*(**F** ^−*^|**H** ^−*^) is shown in blue contours centred on **H** ^−*^ (blue arrow) and the anomalous component *P*(*F* ^+^_obs_|**F** ^−*^, **H** ^+^, **H** ^−^*) is shown in red contours centred on **H** ^+^ − **H** ^−*^, the expected vector difference between **F** ^+^ and **F** ^−*^. The black and red circles indicate the observed structure-factor amplitudes for **F** ^−^ and **F** ^+^, respectively. (*b*) The product of the two components in (*a*) is shown in magenta contours. (*c*) Three-dimensional plot of the value of the PDF under the black circle in (*b*). The likelihood is given as the integral of the height of the surface under the black circle. (*d*) Plot of the likelihood as a function of the occupancy of the substructure (increasing value of |**H** ^−*^| and |**H** ^+^ − **H** ^−*^|). The maximum likelihood is marked with a dot. All other panels in this figure show the values of the parameters at the point of maximum likelihood. (*e*) The PDF for the phases of **F** ^−*^ is shown in magenta and the PDF reconstructed from the four HL coefficients is shown as a black curve. (*f*) Bar chart showing the relative values of the four HL coefficients *A*, *B*, *C* and *D*.

Animated version of Fig. 3.. DOI: 10.1107/S0907444910006335/ba5142sup3.gif

**Figure 4:**
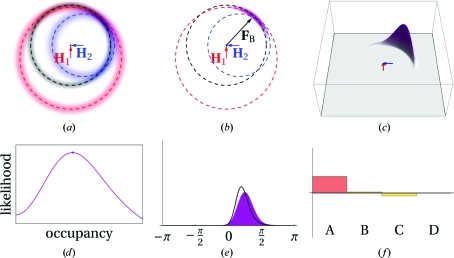
MIR probabilistic Harker diagram (notation as in Fig. 1 ▶). (*a*) Contour plot showing components of the PDF. The component arising from the native is shown in black contours, the component arising from the first derivative is shown in red contours centred on **H**_1_ (the point at the base of the red arrow) and the component arising from the second derivative is shown in blue contours centred on **H**_2_ (the point at the tip of the blue arrow). The dashed black, red and blue circles indicate the measured values of the observed structure-factor amplitudes for the native, first and second derivatives, respectively. (*b*) The PDF [the product of the three components in (*a*)] is shown in dark magenta contours. The ‘best **F**’ **F**_B_ is shown as a black arrow. (*c*) Three-dimensional plot of the value of the PDF. The likelihood is given as the volume under the surface. (*d*) Plot of the likelihood as a function of the occupancy of the substructure for the second derivative (increasing amplitude of **H**_2_). The maximum likelihood is marked with a dot. All other panels in this figure show the values of the parameters at the point of maximum likelihood. (*e*) The PDF for the phases of the true structure factor **F** is shown in dark magenta and the PDF reconstructed from the four HL coefficients is shown as a black curve. (*f*) Bar chart showing the relative values of the four Hendrickson–Lattmanm coefficients *A*, *B*, *C* and *D*.

**Figure 5:**
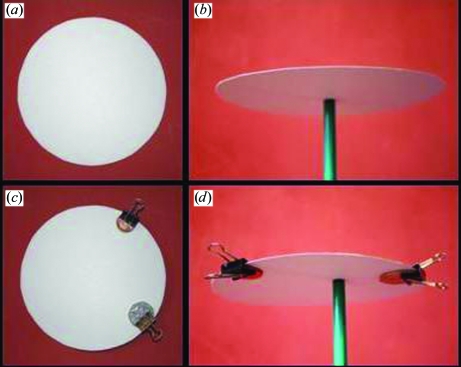
The difference between the ‘centroid’ and ‘most probable’ structure factors. (*a*) Cut the centre out of a paper plate. (*b*) Balance the disc on a pen. The centre of mass is at the centre. (*c*) Now clip two unequal weights to the edge of the plate. (*d*) The balancing point is between the two weights (analogous to the ‘centroid’ structure factor) and not on the heaviest weight (analogous to the ‘most probable’ structure factor).

**Figure 6:**
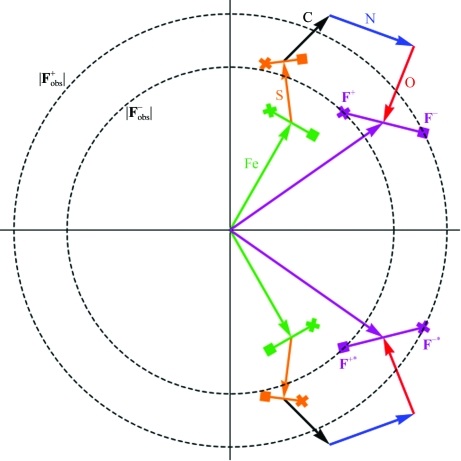
Phasing in both hands. The anomalous scattering component is always advanced. For example, data collected at a wavelength of 1.7 Å from an iron-containing protein will have a significant anomalous signal from both the Fe atoms and the S atoms in methionine and cysteine. Non-anomalous contributions to the scattering come from C, N and O atoms. The total structure factor has an anomalous component that is not perpendicular to the normal scattering component, leading to an anomalous difference in the structure factors for **F** ^+^ and **F** ^−^. Only in one hand will the observed direction of the anomalous difference match the calculated direction of the difference (|**F** ^+^| > |**F** ^−^|).

Animated version of Fig. 4.. DOI: 10.1107/S0907444910006335/ba5142sup4.gif

## Figures and Tables

**Table 1:** Changing the hand of substructure sites For nonchiral space groups the other hand of the heavy-atom sites is found by the operation (*x*, *y*, *z*)→(−*x*, −*y*, −*z*), except for three space groups (*I*4_1_, *I*4_1_22 and *I*4_1_32) where there is also a change of origin. For the chiral space groups the change of hand of the heavy-atom sites with the operation (*x*, *y*, *z*)→(−*x*, −*y*, −*z*) is accompanied by a change of space group to the other chiral form.

System	Chiral	Nonchiral
Triclinic		*P*1
Monoclinic		*P*2, *P*2_1_, *C*2
Orthorhombic		*P*222, *P*222_1_, *P*2_1_2_1_2, *P*2_1_22, *C*222, *C*222_1_, *I*222, *I*2_1_2_1_2_1_, *F*222
Tetragonal	*P*4_1_:*P*4_3_	*P*4, *P*4_1_, *I*4, *I*4_1_†
	*P*4_1_22:*P*4_3_22	*P*422, *P*42_1_2, *P*422, *P*42_1_2, *I*422, *I*4_1_22‡
Trigonal	*P*3_1_:*P*3_2_	*P*3, *R*3
	*P*3_1_12:*P*3_2_12	*P*312, *P*321, *R*32
	*P*3_1_22:*P*3_2_22	
Hexagonal	*P*6_1_:*P*6_5_	*P*6, *P*6_3_
	*P*6_2_:*P*6_4_	
	*P*6_1_22:*P*6_5_22	*P*622, *P*6_3_22
	*P*6_2_22:*P*6_4_22	
Cubic		*P*23, *F*23, *I*23, *P*2_1_3, *I*2_1_3
	*P*4_1_32:*P*4_3_32	*P*432, *P*4_2_32, *F*432, *I*432, *I*4_1_32§

† For *I*4_1_ the origin is shifted to (½, 0, 0).

‡ For *I*4_1_22 the origin is shifted to (½, 0, ¼).

§ For *I*4_1_32 the origin is shifted to (¼, ¼, ¼).

## Appendix A Combined real–imaginary SAD LLG maps

Crystallographers have long appreciated the relationship between the derivative of the target function (generally least-squares in the early days) and the coefficients for a map showing how to improve the model (*e.g.* weighted difference maps, as discussed by Cochran, 1948 ▶). With the replacement of least-squares targets by more powerful likelihood functions, the associated log-likelihood gradient (LLG) maps have proven to be more effective than traditional difference maps in highlighting areas for improvement in the model, such as adding new sites for experimental phasing (de La Fortelle & Bricogne, 1997 ▶).

When anomalous scattering is present, Friedel’s law breaks down for the observed and calculated structure factors and thus also for the derivatives with respect to the calculated structure factors. As a result, an LLG map computed from the derivatives of the log-likelihood target with respect to the calculated structure factors will be a complex-valued function, showing where both real and imaginary scattering should be added to the model to improve the agreement with the data. The real and imaginary components can be inspected as separate residual maps to detect new sites (de La Fortelle & Bricogne, 1997 ▶).

However, we wished to compute maps that identify new sites for particular anomalous scatterers, taking into account the identity of the anomalous scatterer and its characteristic ratio of real and imaginary scattering contributions. We felt that such a map would have two advantages. Firstly, it would integrate the information from both the real and imaginary components and thus reduce the effects of noise. Secondly, it would allow us to distinguish between different types of anomalous scatterer when there is more than one type present in a crystal.

The SAD likelihood target is expressed in terms of **H** ^+^ and **H** ^−*^, where **H** ^−*^ is the complex conjugate of the structure factor for the minus hand. If **U** is a structure factor representing the Fourier transform of the occupancies of a particular anomalous scatterer with the real contribution to its scattering factor given by *f* = *f*_0_ + *f*′ and the imaginary contribution given by *f*′′, then the change in **H** ^+^ and **H** ^−*^ introduced by a change in **U** can be expressed as

We can express these structure factors in terms of their real (*A*) and imaginary (*B*) parts,

and then define the changes in the real and imaginary parts of the calculated structure factors as

If the log-likelihood function is denoted by *L*, an LLG map showing the location of anomalous scatterers can be computed using the coefficients

Applying the chain rule,

The combined real and imaginary SAD LLG maps rely on good estimates of *f*′′, which in *Phaser* are obtained by refinement.

Note that a map computed using *f* = 1 and *f*′′ = 0 will correspond to the real part of a complex-valued LLG map computed from the derivatives with respect to the calculated structure factors and that an LLG map computed using *f* = 0 and *f*′′ = 1 will correspond to the imaginary part of that map. It can be seen from this that the SAD LLG map computed by *Phaser* (McCoy *et al.*, 2007 ▶) gives an appropriately weighted combination of those two components of the complex-valued map. Another way to think of the SAD LLG map is that it is a complex correlation function correlating the complex LLG map with the complex density of a particular anomalous scatterer as a function of translation.

The SAD LLG map will show peaks that are smeared out by the atomic displacements, so we have tested the effect of sharpening, in which the average displacements given by the Wilson *B* factor are removed. In a variety of tests, sharpening sometimes improved the ability of the maps to detect minor sites and never degraded the results. The use of sharpening is the default in *Phaser*.

### A1. Iterative completion

SAD LLG maps show where the likelihood function would like to see changes in the anomalous or heavy-atom model but cannot do anything about changing the model in the current substructure-refinement cycle because there is not (yet) an atom (or other amenable scattering parameter) available for which the scattering can be changed. Adding scattering at peak locations in the SAD LLG maps (and removing scattering from holes) increases the log-likelihood of the model. SAD LLG maps can thus be used to build up (‘complete’) the phasing substructure before beginning any model building that uses stereochemical restraints. This usually requires several iterations, because improvements in the substructure model enhance the sensitivity of the SAD LLG maps to finding minor sites. The algorithm that is iterated until the substructure is stable (converges) in *Phaser* is detailed below.

#### A1.1. Analysis of SAD LLG maps

For each scattering type (and corresponding refined *f*′′) a combined real–imaginary SAD LLG map is calculated as follows.

(i) Selection. Peaks and holes with a *Z* score greater than 6 (default) in the SAD LLG map are selected. To account for particularly noisy maps, peaks with a *Z* score less than that of the deepest hole are also excluded. (Peaks with *Z* scores greater than 6 but less than the *Z* score of the deepest hole may indeed represent true features, but if this is the case the peaks will appear in SAD LLG maps in subsequent cycles of the iterative structure completion and exclusion of a peak by this criterion will only result in an increase in the number of cycles to convergence.)
(ii) Clustering. Peaks and holes are clustered within the separation distance. By default, the separation distance is the maximum of a short bonding distance (1 Å) and the optical resolution of the data (which is equivalent to 71.5% of the high-resolution limit of the data), although the value can also be input by the user. Clustering ensures that atoms will be added with some stereochemical plausibility (in the absence of true bonding criteria).
(iii) *B* swapping. Peaks or holes that are close to atoms of the current substructure with isotropic *B* factors are used to flag these atoms for anisotropic *B*-factor refinement.
(iv) Resurrection. Peaks that are close to atoms of the current substructure that have been rejected in previous cycles (see §[Sec seca1.2]A1.2 below) are used to resurrect the previously rejected atoms.
(v) Potential new sites. Peaks that are not used for either *B* swapping or resurrection are stored for use in defining new atomic sites in site editing (see §[Sec seca1.2]A1.2). The *Z* score of the peak is also stored.

These five steps are repeated for each scattering type (atom type, *f*′′) to be considered for substructure completion. Many peaks will be common to all of the SAD LLG maps; however, their relative weights (*Z* scores) will differ. In order to avoid adding the same site more than once and to select the most probable scattering type, the peaks representing potential new sites from all the SAD LLG maps are clustered (within the separation distance). The peak with the highest *Z* score within each cluster is added as a new site (*i.e.* the position and the scattering type of the peak with the highest *Z* score is used). The scattering type may be altered in a later iteration. Initial values of the occupancy and isotropic *B* factor are taken from their average values for that scattering type already present in the substructure, if applicable; otherwise, the occupancy is set to the expected occupancy and the *B* factor is set to the Wilson *B* factor. The expected occupancy is 0.9, since there is often incomplete incorporation of anomalous scatterers (the data are on an approximate absolute scale).

#### A1.2. Site editing

Independent of the SAD LLG map calculation, the refined substructure (*i.e.* excluding unrefined newly added sites from analysis of the SAD LLG map) is also edited as follows.

(i) Rejection. The current substructure is searched to find atoms that have refined to very low occupancy. The low-occupancy atoms are flagged as ‘rejected’ (but not deleted). If there is a peak near a rejected atom in subsequent cycles then the atom can be resurrected (see §[Sec seca1.1]A1.1). An atom that has been rejected and subsequently resurrected cannot be rejected for a second time: this prevents cycling (infinite loops) of the structure-completion algorithm.
(ii) Change scattering type. The current substructure is also searched to find atoms that have refined to occupancies that deviate greatly from the expected occupancy. These atoms are likely to have been assigned the wrong scattering type, since occupancy and scattering type are highly correlated in SAD refinement. The scattering type is changed to the one that brings the occupancy closest to the expected value. Only those atoms that have been added in previous cycles of structure completion (and not those of the original input substructure) may have their scattering type altered.

### A2. Tests

In tests on structures with more than one type of anomalous scatterer (*e.g.* proteins with iron–sulfur clusters, heavy-atom derivatives with a significant anomalous contribution from intrinsic S atoms, metalloproteins with different metal sites), the SAD LLG maps are considerably better than random at distinguishing between the different types of sites, *i.e.* the map computed for the correct anomalous scatterer tends to give a higher peak (measured by root-mean-square deviations above the mean) than the maps for other anomalous scatterers and the assignment of atom type is usually reliable. When the distinction between atom types is weak, either because of noise in the data or because the ratios of real to imaginary scattering are similar, errors in identifying the correct atom type have little impact on phase quality. Although the distinction between scattering types in the SAD LLG maps (where more than one anomalous scatterer is present) has only a small impact on the overall phase quality, the ability to reliably distinguish the atom types makes it possible to identify the correct hand from the phasing statistics (without the need for density modification) and is very helpful when substructure sites are used as chemical markers in model building.

### A3. Example

The properties of the SAD LLG maps can be illustrated with a test case from a protein containing more than one type of anomalous scatterer. The structure of *Escherichia coli* nitrate reductase A was solved using a combination of Fe-MAD and isomorphous replacement (Bertero *et al.*, 2003 ▶). This protein, which has a molecular weight of about 220 kDa, contains 19 Fe atoms in five Fe–S clusters, two Fe atoms in haem groups, an Mo atom, 118 S atoms (from the Fe–S clusters as well as from cysteine and methionine residues) and five P atoms. We carried out tests using only the peak Fe data, which were collected at a wavelength of 1.7325 Å to a resolution of 2.5 Å. The program *HySS* (Grosse-Kunstleve & Adams, 2003*a* ▶) finds a solution with 11 Fe sites; several of these are actually superatoms representing an entire Fe–S cluster and three are false sites.

When LLG completion is carried out, looking for three atom types (Fe, Mo and S; P was considered to be indistinguishable from S at this wavelength), the final substructure model contains 57 atoms. Of the 49 atoms added to the model in five cycles of completion, 33 are correctly identified from their relative peak heights in the LLG maps, while 16 are misidentified. The reassignment algorithm, which changes the identity of atoms that refine to unusually low or high occupancies, reduces the number of wrongly identified atoms in the final substructure model to six. In the course of refinement and completion all of the superatoms are resolved into individual atomic sites.

Because Friedel’s law is not obeyed for the substructure structure factors when there is a mixture of types of anomalous scatterers, refinement and completion can distinguish between the two possible choices of hand. With the incorrect choice of hand a substructure of only 43 atoms is found and the log-likelihood score is significantly lower than for the correct hand.

The electron-density map obtained with phases from the substructure after completion is of sufficient quality that *ARP*/*wARP* (Cohen *et al.*, 2004 ▶) and *phenix.autobuild* (Terwilliger *et al.*, 2008 ▶) can each trace about 70% of the chain. If the protein model from *ARP*/*wARP* is used as a ‘substructure’ to re-initiate the determination of the anomalous scatterers, the substructure-completion algorithm now finds 105 sites, of which 92 are correctly identified. Such an iterative procedure enhances the phase information and the eventual completeness of the model.

## Footnotes

1 Supplementary material has been deposited in the IUCr electronic archive (Reference: BA5142). Services for accessing this material are described at the back of the journal.
