# Supplementary figures and images for: Experimental phasing: best practice and pitfalls
Source: Acta Crystallogr D Biol Crystallogr. 2010 Mar 24;66(Pt 4):458–69. doi: 10.1107/S0907444910006335 (PMC2852310; doi:10.1107/S0907444910006335)

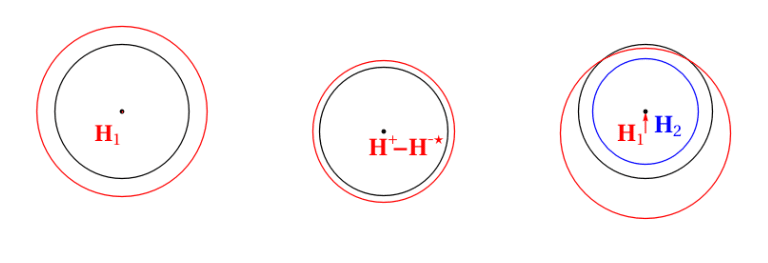

Supplement: Supplementary file 1 [file d-66-00458-sup1.gif]

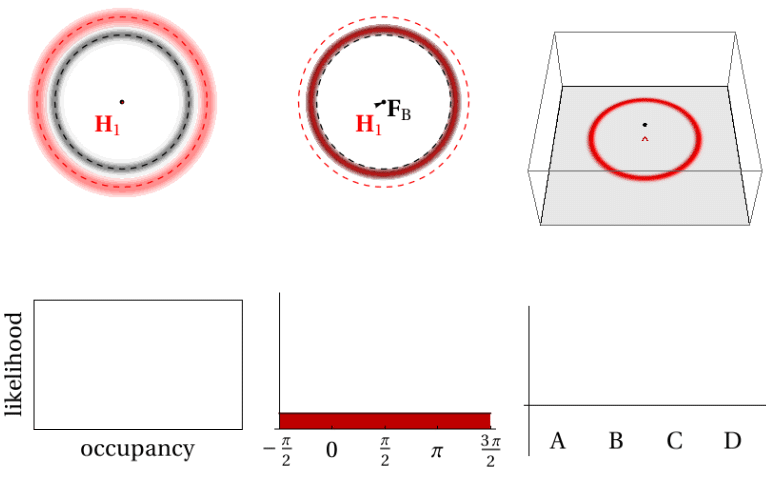

Supplement: Supplementary file 2 [file d-66-00458-sup2.gif]

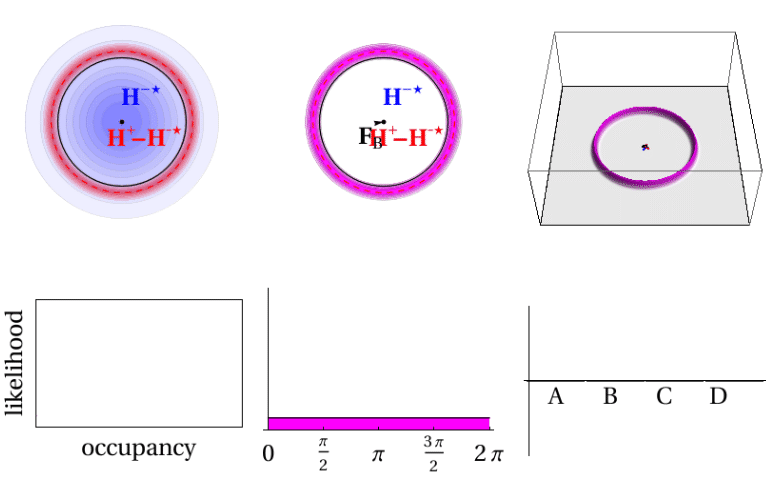

Supplement: Supplementary file 3 [file d-66-00458-sup3.gif]

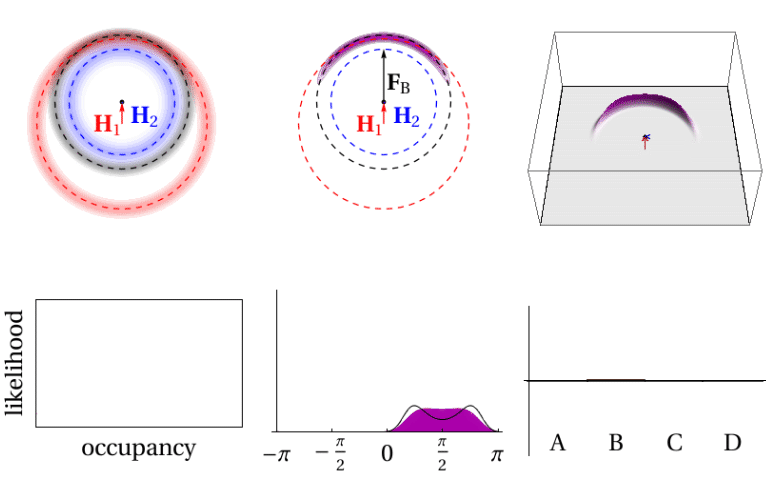

Supplement: Supplementary file 4 [file d-66-00458-sup4.gif]
